# Improving epidemic malaria planning, preparedness and response in Southern Africa

**DOI:** 10.1186/1475-2875-3-37

**Published:** 2004-10-22

**Authors:** Joaquim DaSilva, Brad Garanganga, Vonai Teveredzi, Sabine M Marx, Simon J Mason, Stephen J Connor

**Affiliations:** 1 WHO-ICP-MAL-SAMC; Parirenyatwa Hospital Annex; P.O. Box CY 348 Causeway, Harare, Zimbabwe; 2 SADC Drought Monitoring Centre; P.O. Box 150, Belvedere, Harare, Zimbabwe; 3 Ministry of Health and Child Welfare, P.O. Box CY 1122, Causeway, Harare, Zimbabwe; 4 International Research Institute for Climate Prediction (IRI); The Earth Institute at Columbia University; Monell, Lamont Campus, 61 Route 9W, Palisades, New York 10964-8000, USA

## Abstract

Malaria is a major public health problem for countries in the Southern Africa Development Community (SADC). While the endemicity of malaria varies enormously across this region, many of the countries have districts that are prone to periodic epidemics, which can be regional in their extent, and to resurgent outbreaks that are much more localized. These epidemics are frequently triggered by climate anomalies and often follow periods of drought. Many parts of Southern Africa have suffered rainfall deficit over the past three years and countries expect to see increased levels of malaria when the rains return to more 'normal' levels. Problems with drug and insecticide resistance are documented widely and the region contains countries with the highest rates of HIV prevalence to be found anywhere in the world. Consequently, many communities are vulnerable to severe disease outcomes should epidemics occur.

The SADC countries have adopted the Abuja targets for Roll Back Malaria in Africa, which include improved epidemic detection and response, i.e., that 60% of epidemics will be detected within two weeks of onset, and 60% of epidemics will be responded to within two weeks of detection. The SADC countries recognize that to achieve these targets they need improved information on where and when to look for epidemics. The WHO integrated framework for improved early warning and early detection of malaria epidemics has been recognized as a potentially useful tool for epidemic preparedness and response planning. Following evidence of successful adoption and implementation of this approach in Botswana, the SADC countries, the WHO Southern Africa Inter-Country Programme on Malaria Control, and the SADC Drought Monitoring Centre decided to organize a regional meeting where countries could gather to assess their current control status and community vulnerability, consider changes in epidemic risk, and develop a detailed plan of action for the forthcoming 2004–2005 season. The following is a report on the 1^st ^Southern African Regional Epidemic Outlook Forum, which was held in Harare, Zimbabwe, 26^th^–29^th ^September, 2004.

## Introduction

The Southern African region has a long and varied history of malaria control with periodic epidemics occurring [1,2]. These epidemics can be regional in scale, as in 1996 and 1997, or much more focal, affecting specific districts or sub-districts. The countries of the Southern African Development Community are committed to the Abuja Targets for Roll Back Malaria in Africa, and this includes improved detection and response to epidemics [1]. To meet these targets countries are expected to detect 60% of malaria epidemics within two weeks of onset, and respond to 60% of epidemics within two weeks of their detection. The countries recognize that to achieve these targets they need improved information on where epidemics are most likely to occur, and ideally some indication of when they are likely to occur. The WHO guidelines on the development of Malaria Early Warning Systems (MEWS) for Africa are seen as offering a useful framework for an integrated approach to epidemic preparedness and response planning [3-5]. Experience and evidence of the successful application of this approach within the National Malaria Control Programme in Botswana over the past few years was demonstrated by the national malaria programme manager at the Southern Africa Regional Malaria Planning and Consultation Meeting in Gaborone in July 2004. Other countries in the SADC region considered this approach to provide a useful framework for planning epidemic preparedness and response strategies and, in view of the perceived vulnerability of communities throughout much of the region, called for a regional meeting that could launch the scaling-up of this process to include other epidemic prone countries beyond Botswana. The WHO Southern Africa Inter-Country Programme for Malaria Control (SAMC) responded to this demand and together with SADC's Drought Monitoring Centre (DMC) organized the 1^st ^Southern African Regional Epidemic Outlook Forum, which was held in Harare, Zimbabwe, 26^th^–29^th ^September, 2004 and hosted by Zimbabwe's Ministry of Health and Child Welfare. Representatives from malaria control services in nine Southern African countries participated in the meeting: Angola, Botswana, Madagascar, Mozambique, Namibia, Swaziland, Tanzania, Zambia, and Zimbabwe.

The purpose of the meeting was: to enable malaria control services from epidemic prone countries to gather and review their control programme status and epidemiological trends for the past 3–5 years and identify and map districts they consider to be vulnerable to epidemics; to learn about advances in the science of seasonal climate forecasting and review the implications of the forecast for the forthcoming season; to learn about environmental variables pertinent to epidemic risk and readily available sources of monitoring information; to review methods of early detection using case surveillance data; and, using the WHO framework for MEWS, to develop plans of action for epidemic preparedness and response for the forthcoming season.

## Discussion

**Figure 1:**
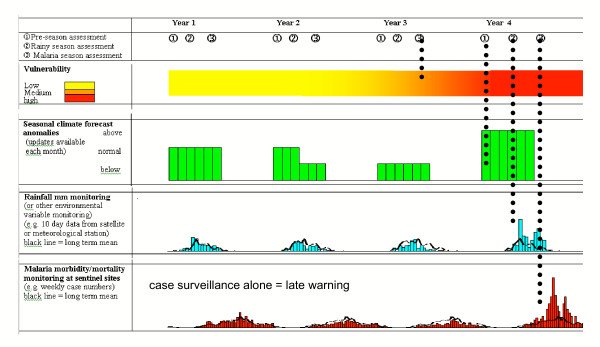
MEWS gathering cumulative evidence for early and focused response (WHO 2004)

The MEWS framework as set out by WHO consists of four components: 1) vulnerability monitoring; 2) seasonal climate forecasting; 3) environmental monitoring; and 4) sentinel case surveillance. This framework is illustrated in Figure 1.

### Vulnerability monitoring

There are many factors that increase the vulnerability of a population to malaria epidemics [6,7] and increase the severity of disease outcome should a malaria epidemic occur. Co-infection with other diseases such as HIV-AIDS is a major consideration for Southern African countries. Resistance to therapeutic drugs and insecticides has also been a recent problem throughout much of the region. Drought, food insecurity and associated population movements between areas of differing endemicity combine to make certain populations more vulnerable to epidemics. These factors and consideration of the where and how to get appropriate information were discussed and countries were encouraged to identify measurable indicators and key informants.

### Seasonal climate forecasting

In recent years there have been significant scientific advances in our ability to predict climate on the seasonal timescale [8]. The skill associated with these predictions varies from region to region, but is generally higher within the tropics. Scientists from the SADC Drought Monitoring Centre and the International Research Institute for Climate Prediction (IRI) joined with meteorologists from Democratic Republic of Congo, Malawi, Namibia, Zimbabwe and the World Meteorological Organization (WMO) to deliver the climate forecast for the forthcoming 2004–2005 season. An overview of climate variability in the SADC region was presented. The inherent issues of probability and uncertainty in climate forecasting were discussed with participants from the malaria control services. A number of myths were exploded and the variables that could or could not be skilfully forecast were reviewed. The malaria control participants gave their views on how communication of the forecast should be improved and made more understandable to the non-climate-specialist. Following a subsequent working session by the climate and meteorological specialists, an outline of additional or alternative forecast indicators was provided.

### Environmental monitoring

The availability of environmental variables pertinent to malaria transmission, such as rainfall, temperatures, humidity, and flooding, were discussed and information on where they could be obtained was provided. The two basic sources of such information are periodic summaries (usually satellite-derived and interpolated estimates) available through the internet from the SADC DMC, the Famine Early Warning Systems Network (FEWS-NET) or the International Research Institute for Climate Prediction, Columbia University (IRI) websites; or directly from national meteorological services' ground-based weather observations. Generally, summary products are available free of charge, whereas the meteorological services may need to charge for raw data. Countries were encouraged to begin dialogue with their national meteorological services and discuss the more specific information requirements and support they may need.

### Sentinel case surveillance

The paramount importance of developing good health information and sentinel surveillance systems was acknowledged. The process of MEWS development is seen as offering opportunities for strengthening integrated health systems surveillance. It is in itself dependent on good epidemiological data for testing and validating the relationships between the component parts. Methods of using indicators for epidemic early detection were discussed. Various indicators such as the mean × 2 standard deviations, the 'normal channel', cumulative-sum and weekly case thresholds have been tried, tested and used in a number of Southern Africa countries, and countries are encouraged to develop and use what is most appropriate and effective for their purposes. However, a number of the countries acknowledged having a poor statistical basis on which to develop and test early warning and detection indicators.

Following the formal presentations setting out the MEWS components and epidemiological trends, the discussions centred around the countries' perceived control needs over the coming season and the information requirements for developing appropriate plans of action for epidemic preparedness and response. The countries represented varied markedly in their current levels of endemicity/epidemicity, surveillance and control coverage. Tanzania is for the most part a highly endemic country with an estimated 16–19 million cases per year. Botswana and Swaziland, by contrast, are currently recording cases in the low thousands and hundreds respectively. Zimbabwe's economic situation has recently compromised its control programme, and two of the countries, Mozambique and Angola, are in process of reconstructing their control programmes after recently emerging from major disruption due to long-term conflict situations. However, all of the countries did acknowledge the integrated MEWS approach as offering a useful framework for improving their epidemic planning, preparedness and response capabilities.

Based on the the climate forecast for October, November, December, and the extended forecast for January, February, March, which are posted on the DMC website . The participants discussed the difficulties in access to and interpretation of meteorological data. The representatives from the meteorological services expressed a willingness to engage in closer collaboration to address these issues. The participants voiced a clear need to improve the availability of the seasonal climate forecast to the epidemic prone districts. They also highlighted the need for better communication of the forecast to non-climate users. Requests were specifically made for forecasts that are more 'meaningful' to the health sector. In response, the meteorological sector pointed at the necessity to know more specifically what information the health sector requires in order to then meet this need. Forecasts could, for example, be expressed simply as the probability of the coming season being wetter or drier than the previous season, or two, or three, or *n *seasons; or compared to that of the last epidemic season; or as probabilities of exceeding a given threshold for the season. However, it was stressed that forecasts will always be probabilistic and not deterministic. Moreover, countries were encouraged to refer to forecast updates as the season progresses. The issues of how to communicate better the probabilities and uncertainties associated with seasonal climate forecasts were addressed more closely. While many activities in malaria control are based on probabilistic, uncertain premises (clinical diagnosis and presumptive treatment, for instance), public health professionals are well aware of the limitations of their own indicators. While recognizing the potential value of advance lead-times for planning, they are understandably cautious in basing critical decisions on uncertain information from others, and the health and meteorological sectors probably need to work this through in more local collaborative settings.

One additional issue that came out strongly during the meeting was the need for broader cross-border collaboration on epidemic prevention and control as 'true epidemic' prone areas are often based on particular environmental zones rather than administrative boundaries. For example, high rainfall anomalies in Angola may ultimately find their way as increased stream-flow into Botswana and Namibia, and create extensive breeding sites for vectors. Drought, food security, or a range of other factors, may lead to migrations of people across borders from one level of endemicity to another and pose a significant increase in epidemic risk. Development of national epidemic risk maps therefore ought to reflect the situation in neighbouring countries. There are a few examples of cross-border initiatives in the region: Republic of South Africa, Swaziland and Mozambique; Republic of South Africa and Zimbabwe. Both are showing promising results.

## Conclusions

The meeting ended with the presentation of the recommendations, to be followed up within the next twelve months. The majority of the recommendations highlighted the need for stronger collaboration a) within the health sector itself; b) among the health sector and the climate-meteorological sector, and other relevant sectors; and c) among the various countries in the region. The participants committed themselves, with their partners, to developing integrated early warning systems as a decision support tool for improving epidemic preparedness and response planning. They recognized that this will be best achieved by drawing on appropriate scientific and technological advancements (and challenging these where necessary), by conducting operational research, and with the help of technical development and support, strengthen the capacity for improved epidemic preparedness and response in the districts at risk.

The successful implementation of MEWS will depend on close cooperation among several partners: National Malaria Control Programmes must work closely with National Meteorological Services, supported by the regional Drought Monitoring Center, WMO and WHO. Opening these channels of communication will allow public health professionals and climate-environmental scientists and practitioners to incorporate more meaningful variables into the seasonal forecasts. In addition, it is necessary to exchange information with institutions dealing with vulnerable populations such as food security and refugee agencies, to develop mutually beneficial mechanisms that ensure easy access and utilization of relevant information for planning or decision-making. It was recognized that by adopting the MEWS approach for malaria control planning the overall health information and surveillance system would be strengthened as other diseases have strong climate and environmental components to their distribution, and further dialogue with Integrated Disease Surveillance and Response services would be useful. Training and capacity building requirements were discussed with WHO-AFRO regarding implementation within the national IDSR activities in the Southern Africa sub-region, and sub-regions elsewhere in Africa. In the final evaluation of the meeting participants, from both health and climate communities, considered that the meeting had provided a very useful overview of the issues and offered a good starting point for them to develop more flexible Plans of Action for Epidemic Preparedness and Response in their countries. It was recommended that a similar meeting be held each year prior to the onset of the rainy season.

## References

[B1] SADC (2003). SADC Malaria Report 2003. Gaborone: Southern Africa Development Community.

[B2] Musawenkosi L, Mabaso H, Sharp B, Lengeler C (2004). Historical review of malarial control in southern Africa with emphasis on the use of indoor residual house-spraying. Trop Med Int Health.

[B3] WHO (2001). Malaria Early Warning Systems: concepts, indicators and partners: A framework for field research in Africa.

[B4] WHO (2004). Malaria epidemics: forecasting, prevention, early warning and control – From policy to practice.

[B5] Thomson MC, Connor SJ (2001). The development of malaria early warning systems for Africa. Trends Parasitol.

[B6] Bates I, Fenton C, Gruber J, Lalloo J, Lara AM, Squire SB, Theobald S, Thomson R, Tolhurst R (2004). Vulnerability to malaria, tuberculosis, and HIV/AIDS infection and disease: Part II: determinants operating at environmental and institutional level. Lancet Inf Dis.

[B7] Bates I, Fenton C, Gruber J, Lalloo J, Lara AM, Squire SB, Theobald S, Thomson R, Tolhurst R (2004). Vulnerability to malaria, tuberculosis, and HIV/AIDS infection and disease: Part 1: determinants operating at individual and household level. Lancet Inf Dis.

[B8] Goddard L, Mason S, Zebiak S, Ropelewski R, Basher R, Cane M (2001). Current approaches to seasonal to interannual climate predictions. Int J Climatol.

